# Genomic signatures of artificial selection in fecundity of Pacific white shrimp, *Penaeus vannamei*

**DOI:** 10.3389/fgene.2022.929889

**Published:** 2022-08-29

**Authors:** Juan Sui, Sheng Luan, Jiawang Cao, Ping Dai, Xianhong Meng, Kun Luo, Baolong Chen, Jian Tan, Qiang Fu, Jie Kong

**Affiliations:** ^1^ Key Laboratory for Sustainable Utilization of Marine Fisheries Resources, Ministry of Agriculture, Yellow Sea Fisheries Research Institute, Chinese Academy of Fishery Sciences, Qingdao, China; ^2^ Laboratory for Marine Fisheries Science and Food Production Processes, Qingdao National Laboratory for Marine Science and Technology, Qingdao, China

**Keywords:** fecundity, whole-genome analysis, single nucleotide polymorphisms (SNPs), *Penaeus* vannamei, selective sweep analysis

## Abstract

*Penaeus* *vannamei* is the most important economic shrimp in the world. Many selective breeding programs are carried out to improve its production and performance traits. Although significant differences in the reproductive ability of female *P. vannamei* under artificial breeding conditions have been reported, the genome-wide adaption of the reproductive ability of domesticated female *P. vannamei* is less investigated. In this study, whole-genome analysis was performed along with pooled DNA sequencing on two fecundity separated bulks, high fecundity bulk (HB), and low fecundity bulk (LB). Each bulk contained 30 individuals from 3 commercial populations. A sequencing depth of >30× was achieved for each bulk, leading to the identification of 625,181 and 629,748 single nucleotide polymorphisms (SNPs) in HB and LB, respectively. Fixation index (Fst) combined with *p* ratio allowed for the identification of 145 selective sweep regions, with a sequence length of 14.5 Mb, accounting for 0.59% of the genome. Among the 145 selective sweep regions, a total of 64,046 SNPs were identified, and further verification was performed by genotyping 50 candidate SNPs on 60 samples from the offspring of the three populations. Furthermore, 121 genes were screened from the sweep regions. GO annotation and KEGG enrichment analyses showed that partial genes were essential for fecundity regulation. This study provides important information for in-depth investigation of genomic characteristics for long-term selective breeding on the fecundity of female *P. vannamei* and will also be important for genome-assisted breeding of *P. vannamei* in the future.

## Introduction

Pacific white shrimp (*Penaeus vannamei*) is the most important economic shrimp in the world, with a global aquaculture production of 5,446,216 tons and a value of 32,190,978 dollars in 2019 (FAO, 2022). The breeding of *P. vannamei* has lasted for 30 years and made great achievements in growth-related traits, survival, and specific disease resistance (Benzie, 2009; Gjedrem, 2012; Castillo-Juárez et al., 2015; Gjedrem and Rye, 2016).

The reproductive performance of parent shrimp is an essential trait for commercial larval production (Arcos et al., 2004; Caballero-Zamora et al., 2015). However, there are significant differences in the fecundity of female *P. vannamei* in seedling production. In a production cycle, some females can spawn up to 15 times, whereas some other females never spawn (Arcos et al., 2003; Tan et al., 2019). To improve larval production, selecting female shrimp with high fecundity is a vital strategy (Racotta et al., 2003; Arcos et al., 2004). However, knowledge about the genetic basis of such significant differences in the reproductive traits of female *P. vannamei* is very limited.

Both artificial breeding and directional selection would gradually affect the phenotype of domesticated animals, which will lead to the abundant phenotypic diversity and genetic adaptability of domesticated animals (Andersson, 2012 and 2013). The process of artificial breeding would leave detectable signatures on the genomes of domestic species (Utsunomiya et al., 2015; López et al., 2019). Analyzing these signatures is critical to study the genomic regions that changed due to domestication and genetic improvement.

Whole-genome resequencing has become a practical approach for the analysis of genome-wide variations and genomic signatures of domestication or artificial selection (Stratton, 2008; Rubin et al., 2010; Xia et al., 2015; Zhang et al., 2018). The method has been applied to many aquatic animals, such as Atlantic salmon (Bertolotti et al., 2020), Nile tilapia (Cadiz et al., 2019), channel catfish (Nam et al., 2020), large yellow croaker (Kon et al., 2021), and black tiger shrimp (Wong et al., 2020). Such analyses are usually costly because a large number of individuals need to be genotyped individually. In the population with a wide variation of target traits, selecting extreme or representative individuals can significantly reduce the scale and cost. The DNA pooling approach has been widely used to study genomic signatures of artificial selection in animals (Bertolini et al., 2016; Naval-Sanchez et al., 2020).

Fecundity is one of the noninvasive predictive criteria to identify females with high spawning frequency (Arcos et al., 2003). However, there are still no studies focusing on the identification of selection signatures in the reproductive traits of farmed Pacific white shrimp populations to date. In this study, we focused on the egg-laying frequency (ELF) and average egg amount of female parent *P. vannamei* in a production cycle. To increase the knowledge of selection effects on female reproduction in farmed populations of this species, selective sweeping analysis was performed on two fecundity separated bulks of *P. vannamei* using whole-genome resequencing with pooled DNA sequencing (DNA-seq). Single nucleotide polymorphism (SNPs) and genes associated with reproductive traits were identified. This work will contribute to the deep investigation of the molecular mechanisms of reproduction and the development of molecular breeding for high reproductive traits of *P. vannamei*.

## Materials and methods

### Animals

In 2018, three commercial populations of *P. vannamei* were introduced to BLUP Aquabreed Co., Ltd (Weifang, Shandong Province, China). The three populations that contained 65 families were selected for years and labeled A, B, and C, respectively, among which A and B were characterized by strong resistance and C by fast growth. In 2019, the shrimps were cultivated as candidate parents. By the age of 10 months, 8–10 females and 8–9 males from each family were selected. Females subjected to unilateral eyestalk ablation to promote the synchronous development of ovaries and a number-coded ring were placed on the remaining eyestalk. At the same time, the parent shrimp started to undergo nutrition fortification for about 20 days. A total of 604 females and 500 males underwent nutrition fortification (Table 1). The diet consisted of two meals of squid and two meals of *Nereis* for females and three meals of squid and one meal of *Nereis* for males, which was accounted for a total daily supply of 20% of wet weight biomass, and was adjusted daily. Females were cultured in six 16-m^2^ tanks with a density of six to seven shrimp/m^2^. Males from the same family were placed in a 3-m^2^ tank, with a density of two to three shrimp/m^2^.

**TABLE 1:** The composition of high- and low-reproductive bulks.

Population	Total family	Total females	Females with offspring	Family	HB individual	HB Family	LB individual	LB family
A	14	142	35	13	13	9	12	9
B	26	206	37	22	10	9	7	5
C	25	256	15	8	7	5	11	7
Total	65	604	87	43	30	23	30	21

HB, high-reproductive bulk.

LB, low-reproductive bulk.

### Reproductive traits recording

The next generation was produced by mating selected male and female families at a ratio of 1:2 to 1:5, imposing certain restrictions for controlling inbreeding (e.g., avoiding full-sib, half-sib, and cousin mating). Sexually matured females were placed into the tank with males for natural mating at 9:00 a.m. and collected at 7:00 p.m. every day (recording the female shrimp number-coded ring). The mated females were put into the 120-L spawning tank (recording number-coded ring), whereas the unmated females were put back to the original tank. Male shrimp of one family mated with female shrimp from two different families at most at 1 day. The family construction period was from September 4 to 29 September 2019.

The ELF of 604 females during this period and the average egg number (AEN) from each spawn per female was recorded. Among these individuals, 179 had spawning records and 87 had offspring that successfully developed to juvenile stage. We only selected high reproductive individuals from the 87 females with offspring, because the vitality of offspring can also reflect the reproductive performance of females to some extent, although not absolutely. According to the ELF and AEN, we developed a fecundity index to measure the fecundity of female shrimp. The calculation formula was as follows:

$$y_{i}=0.5*\left(a_{elfi}-\mu_{elf}\right)*\sigma_{elf}^{-1}+0.5*\left(a_{aeni}-\mu_{aen}\right)*\sigma_{aen}^{-1}$$

where *y*_*i*_ is the fecundity index of the *i*th individual of the 87 spawning individuals; *a*_*elfi*_ and *a*_*aeni*_ are the ELF and AEN of the *i*th individual, respectively; *μ*_*elf*_ and *μ*_*aen*_ are the average of the ELF and AEN of the 87 spawning individuals; and *σ*_*elf*_ and *σ*_*aen*_ are the standard error of the ELF and AEN of the 87 spawning individuals.

According to the reproductive index, a total of 30 females with high reproductive index and 30 individuals with no sexual maturity record were selected from the three populations to comprise a high reproductive bulk (HB) and a low-reproductive bulk (LB), respectively.

Muscle samples of 30 high- and 30 low-reproductive shrimp were collected and frozen in liquid nitrogen. For genotyping, the total genomic DNA of each individual was isolated using cetyltrimethylammonium bromide (CTAB). Equal DNA content quantified by Qbit 2.0 (Invitrogen, CA, United States) was pooled separately to generate a “high” pool and a “low” pool.

### High-throughput sequencing and clean read filtering

Paired-end sequencing libraries with an insert size of about 500 bp were constructed using the Paired-End DNA Sample Prep Kit (Illumina Inc., San Diego, CA, United States). The libraries were sequenced on an Illumina HiSeq 2500 system using standard protocol (Illumina, Inc.; San Diego, CA, United States). The sequence depth of two pools was 30× on average.

Raw reads were processed to obtain high-quality clean reads according to three stringent filtering standards: 1) removing reads with ≥10% unidentified nucleotides (N); 2) removing reads with >50% bases having Phred quality scores of ≤20; and 3) removing reads aligned to the barcode adapter.

### Variant identification and annotation

To identify SNPs, the Burrows–Wheeler Aligner (BWA, Li and Durbin, 2009) was used to align the high-quality reads from each pool against the reference genome (GenBank accession numbers QCYY01000001-QCYY01004682; Zhang et al., 2019) with the settings “mem 4-k 32-M.” Variant calling was performed using the GATK’s UnifiedGenotyper. SNPs were filtered using GATK’s Variant Filtration with proper standards (-Window 4, -filter “QD < 2.0 || FS > 60.0 || MQ < 40.0,” -G_filter “GQ < 20”), and those exhibiting segregation distortion or sequencing errors were discarded. To determine the physical positions of each SNP, the software tool ANNOVAR (Wang et al., 2010) was used to align and annotate SNPs. Insertions/deletions (indels) were not considered in this study because the reliability of next generation sequencing to detect such variants was relatively low (Boland et al., 2013).

### Selective sweep analysis

Selective sweep regions were selected according to the interception of two parameters, Fixation Index (Fst) (Hudson et al., 1992) and *p* ratio (ratio of nucleotide diversity between two populations, πA/πB) (Nei and Li, 1979). A 50-kb sliding window approach with 25-kb step size was applied to quantify these parameters with in-house PERL scripts. Regions under selection were identified using an empirical procedure (Li et al., 2018). In brief, genomic regions with significantly biased *p* ratios (*p* < 0.01) and significantly high Fst values (*p* < 0.01) of the empirical distribution were regarded as regions with strong selection signals along the genome. All related graphs were drawn using R scripts (R Core Team, 2006).

Candidate SNPs and genes within the sweep regions were extracted for further analysis. To evaluate the reliability of SNP prediction using pooled DNA-seq data, 50 candidate SNPs were genotyped in 60 samples based on individual DNA-seq data using the MALDI-TOF method. The 60 samples were obtained from the offspring of the three populations with contrasting reproduction traits, among which 30 were of high fecundity and 30 were of low fecundity. The SNP index of each locus and the frequency difference distribution in the two bulks were calculated. The direction was as follows: Δ(index) = index (HB) − index (LB). Only significant SNPs were selected as candidates for validation. The locus with Δ index close to 1 or to −1 was used as the preferred sites for verification.

All candidate genes were mapped to GO terms in the Gene Ontology database (http://www.geneontology.org/). Gene numbers were calculated for every term, and significantly enriched GO terms were defined using hypergeometric test with *p* < 0.05. KEGG is a major public pathway–related database. The calculation formula is the same as that in GO analysis. Pathways with *p* < 0.05 were defined as significantly enriched pathways in genes.

## Results

### Bulk segregation for reproduction

**FIGURE 1:**
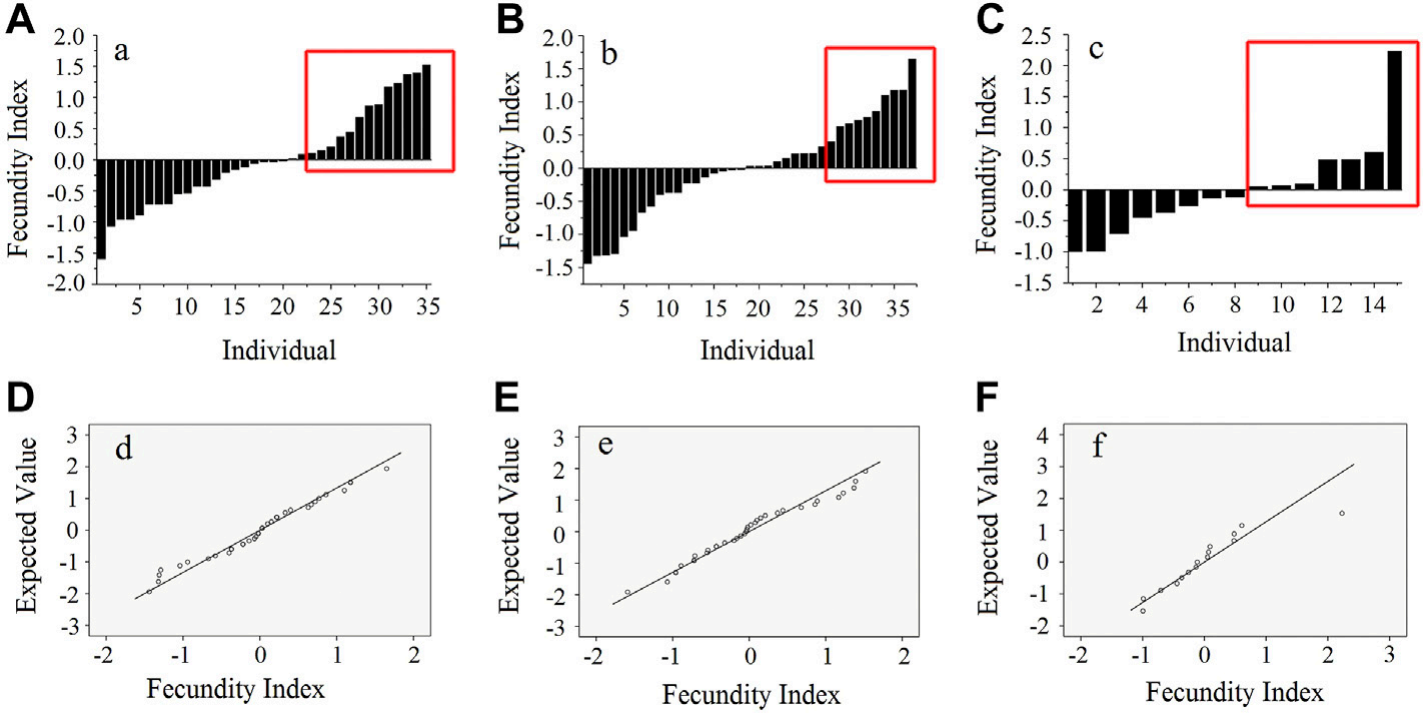
Distribution of fecundity indices. **(A–C)** Fecundity indices of 87 females with offspring in population. **(A–C) (D–F)** The QQ plot shows a normal distribution of females with offspring in three populations.

The fecundity indices of 87 females with offspring ranged from −1.59 to 1.52 for population A, −1.44 to 1.65 for population B, and −0.99 to 2.23 for population C (Figures 1A–C). In the QQ plots, most points were close to the fitting line, which showed that the fecundity indices of those females displayed normal distribution patterns in the three populations after the Shapiro–Wilk tests (*p* > 0.05, Figures 1D–F). In populations A, B, and C, 13, 10, and 7 individuals with the highest fecundity index were selected into HB, respectively. The average value of the fecundity indices of HB were 0.80, 0.91, and 0.57, respectively. In populations A, B, and C, 12, 7, and 11 individuals with no sexual maturity record were selected into LB, respectively (Table 1; Figures 1A–C). There were nine mutual families between HB and LB.

### Identification of chromosome regions related to fecundity

Using Illumina resequencing, a total of 1,192 million reads were obtained with about 548.87 million reads from HB and 643.51 million reads from LB (Table 2). Raw read data of HB and LB were deposited in the NCBI Sequence Read Archive under the accession number PRJNA610662. After quality control, 1,106 million reads were used for further analysis, giving rise to total clean data of 137.89 Gb with an average read length of 150 bp.

**TABLE 2:** Summary of Illumina sequencing of the high fecundity bulk (HB) and low-fecundity population (LB).

	HB	LB	Total
Number of reads	548,872,514	643,514,314	1,192,386,648
Number of reads after filtering	510,897,932	595,946,642	1,106,844,574
Percentage kept after filtering (%)	93.08	92.61	92.83
Clean Q30 bases Rate (%)	78.95	75.49	77.09
Genome coverage	30.40 ×	30.47 ×	60.87 ×

**FIGURE 2:**
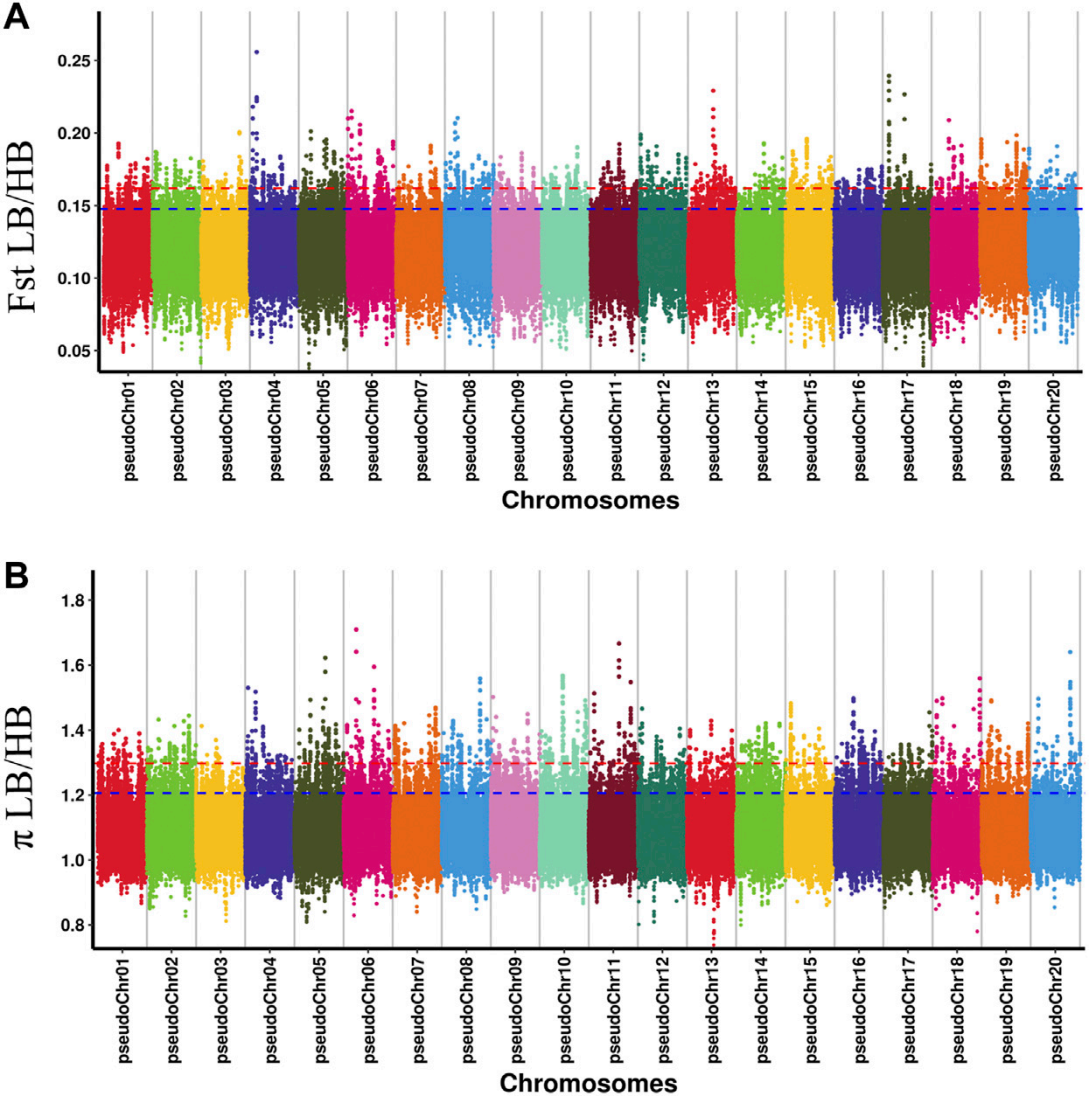
Manhattan distribution of fixation index (Fst) **(A)** and *p* ratio **(B)** of each pseudochromosome.

The Fst and *p* ratio were used to detect the selective sweep signals between HB and LB (Figure 2). The thresholds of each index determined by *p* < 0.01 were as follows: 0.1619 for Fst and 1.2977 for *p* ratio. The intersection part was considered as the reliable candidate interval. The significant interval and gene information in the interval were obtained according to the above threshold. The candidate region was generally the chromosome region with a high degree of genetic differentiation between HB and LB.

**FIGURE 3:**
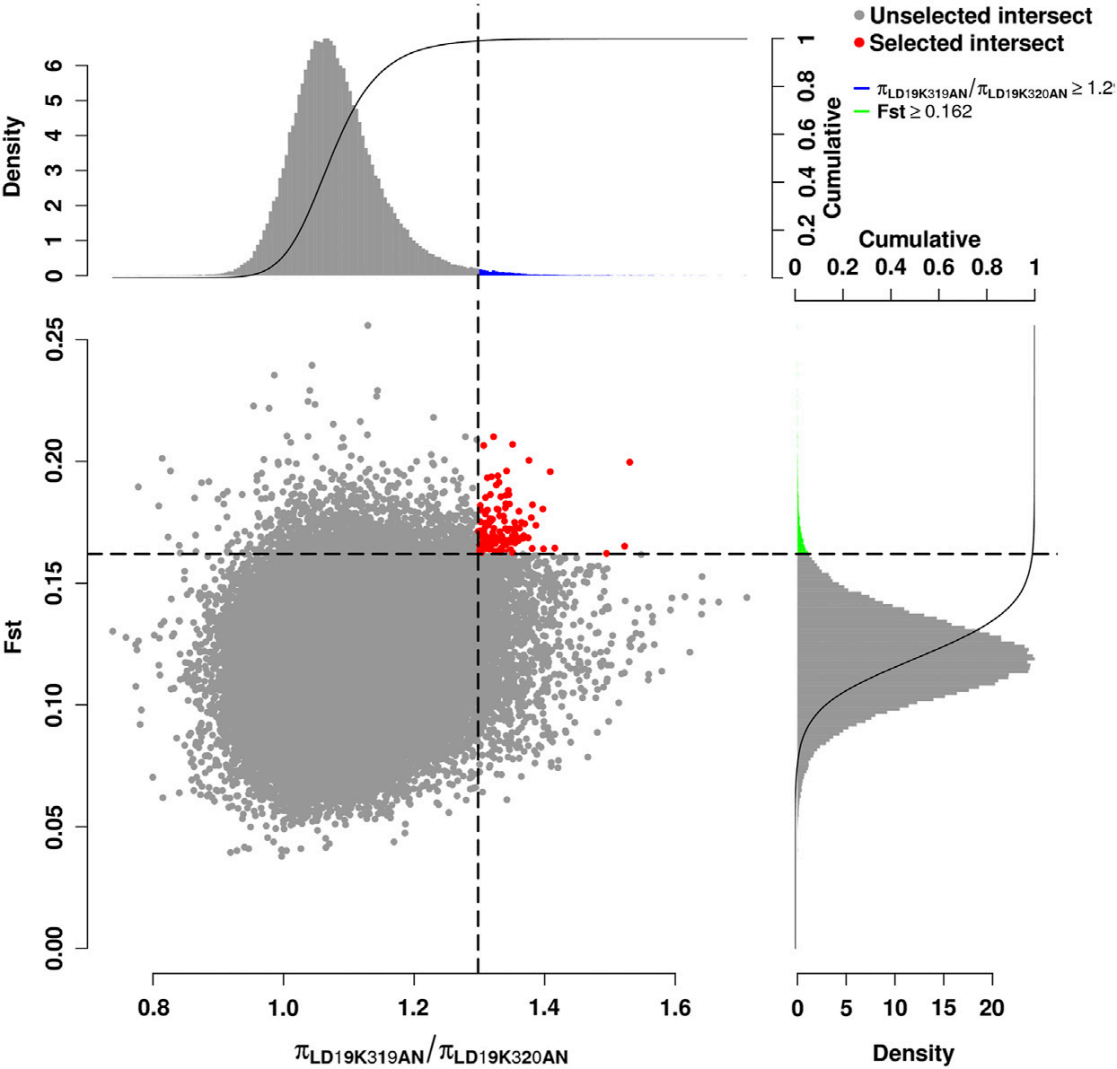
Schematic diagram of selection signal.

The intersection of selected parts of the Fst and *p* ratio is presented in Figure 3. The upper and bottom panels are the Fst and *p* ratio, respectively, for 100-kb windows compared between HB and LB. A total of 145 selected regions were screened, with a sequence length of 14.5 Mb, accounting for 0.59% of the genome.

### Identification and validation of single nucleotide polymorphisms within sweep regions

**FIGURE 4:**
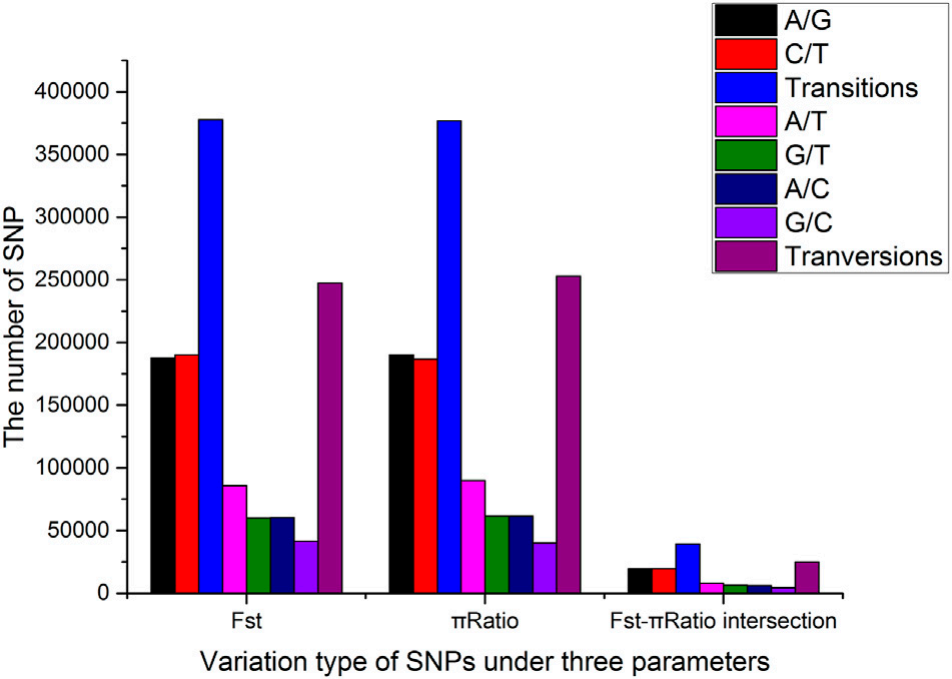
Type distribution of single nucleotide polymorphisms (SNPs) shared by the high fecundity bulk (HB) and low-fecundity population (LB) under two parameters.

All significant SNPs were assigned to 20 tentative chromosomes based on the *P. vannamei* genome sequence (GenBank accession number: PRJNA508983). A total of 625,181 SNPs were shared by HB and LB based on Fst, and 629,748 SNPs were shared based on the *p* ratio (*p* < 0.01). Among them, 377,714 and 376,872 were transitions (Ts) and 247,467 and 252,876 were transversions (Tv), with Ts/Tv ratios of 1.53 and 1.49, respectively (Figure 4). After the intersection of the limited SNP ranges of the two parameters, 64,046 significant SNPs were found, among which 1,136 were located in the coding regions, 12,048 in the intronic regions, 1,651 in the upstream or downstream regions, 49,187 in the intergenic regions, and 24 in the splicing regions.

To validate the reliability of SNP prediction using selected sweep analysis, 50 significant SNPs were genotyped in 60 independent samples based on their respective DNA. After removing the loci with a genotyping completion rate of less than 80%, there were 44 effective loci left. All the 44 candidate SNPs were polymorphic in HB, and 42 candidate SNPs were polymorphic in LB. The allele frequency distributions of these SNPs in HB and LB are presented in Supplementary Table S2. Six loci showed significant allelic imbalance between HB and LB at the level of *p* < 0.05, among which two loci were at the level of *p* < 0.01 (Table 3).

**TABLE 3:** Comparison of allele frequency distributions of 44 candidate single nucleotide polymorphisms (SNPs) between the HB and LB using Pearson’s chi-squared test.

SNP ID	Allele	Allele frequency		*p*
		HB	LB	
X1W1	T	51.67	26.67	0.005
	G	48.33	73.33	
7W1	A	46.67	70.00	0.010
	G	53.33	30.00	
9W1	A	80.00	63.33	0.043
	G	20.00	36.67	
27W1	A	1.67	16.67	0.004
	G	98.33	83.33	
7W2	C	7.14	0.00	0.035
	G	92.86	100.00	
21W2	A	36.67	56.67	0.028
	G	63.33	43.33	

### Identification and functional annotation of candidate genes within sweep regions

**FIGURE 5:**
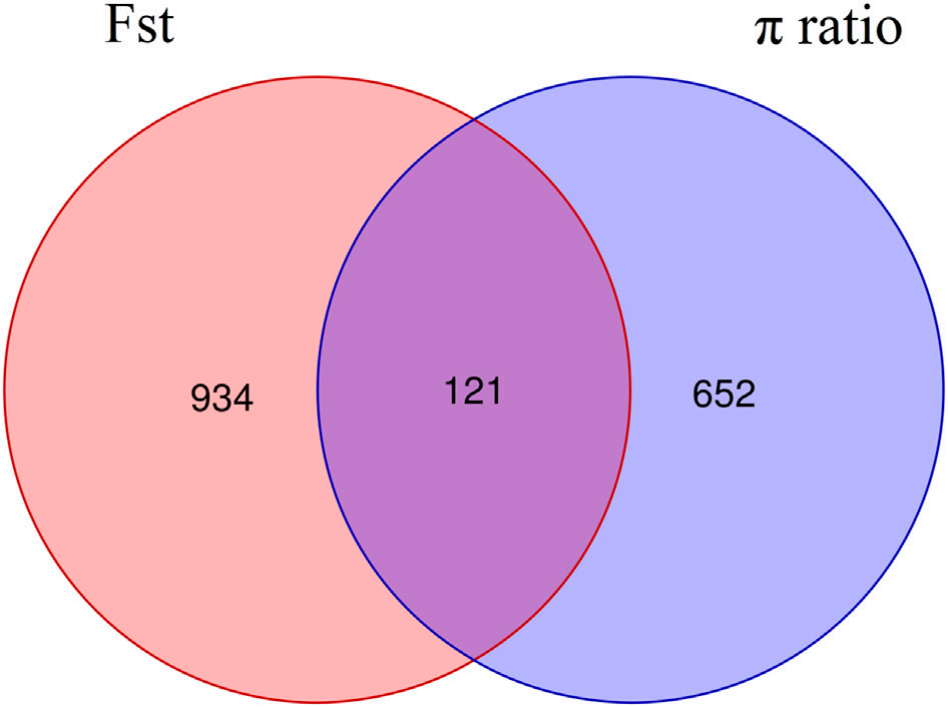
Venn diagram of genes in the selected regions screened by Fst and *p* ratio.

**FIGURE 6:**
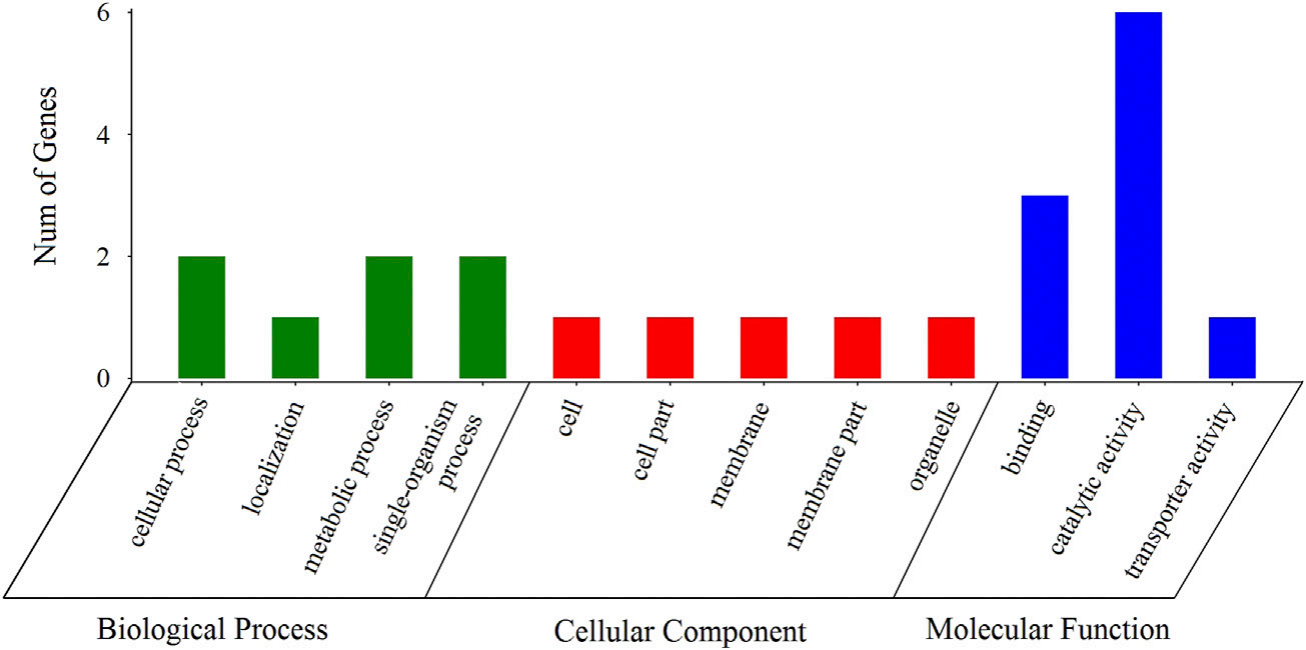
Distributions of candidate genes in different GO categories.

Among the 145 selective sweep regions, 121 genes were identified (Figure 5, Supplementary Table S1). These genes provided genomic evidence of selection signatures and could be used as important loci for targeted genotyping in shrimp breeding. Thus, these genes are interestingly functional candidates for reproduction-related traits under selection on these populations. To further analyze the functions of these genes, we carried out GO annotation and KEGG enrichment analyses. As presented in Figure 6, the abscissa represented the three ontologies of GO: molecular function, including cellular process, localization, metabolic process, and single-organism process; cellular component, including cell, cell part, membrane, membrane part, and organelle; and biological process, including binding, catalytic activity, and transporter activity. Most of the highly enriched GO terms were associated with biological process and molecular function (Supplementary Table S3). The genes related to molecular function were included in the cyclohydrolase (GO: 0019238), acetylglucosaminyltransferase (GO: 0008375), and hydroxymethyl-, formyl-, and related transferase (GO: 0016742) activities. The genes related to biological process were included in calcium ion transport (GO: 0006816), divalent metal ion transport (GO: 0070838), divalent inorganic cation transport (GO: 0072511), and purine nucleotide metabolic process (GO: 0006163). Many genes were categorized into cellular and metabolic processes. Both categories had cell growth or anabolic/catabolic process resulting in cell growth, suggesting that these genes could be associated with fecundity in shrimp.

KEGG analysis showed that the 121 genes were enriched into 73 pathways, among which 19 pathways with *p* < 0.05 and 15 pathways with *Q* < 0.05 (adjusted *p-*value) were identified as significantly enriched pathways (Table 4, Supplementary Table S4). Most of the highly enriched pathways included cardiac muscle contraction (ko04260), adrenergic signaling in cardiomyocytes (ko04261), and cGMP–PKG signaling pathway (ko04022). The KEGG analysis showed that the pathways of some genes have been proven to be closely related to ovarian development, such as serotonergic synapse (ko04726) (Jayasankar et al., 2020). Some of the 121 candidate genes related to fecundity are presented in Table 5.

**TABLE 4:** Detailed information of significantly enriched KEGG pathways of the candidate genes.

Pathway ID	Pathway	Genes with pathway annotation (28)	*p*-value	*Q*-value^a^
ko04260	Cardiac muscle contraction	8 (28.57%)	0.000000	**0.000000**
ko04261	Adrenergic signaling in cardiomyocytes	9 (32.14%)	0.000000	**0.000000**
ko04022	cGMP - PKG signaling pathway	9 (32.14%)	0.000000	**0.000000**
ko04919	Thyroid hormone signaling pathway	9 (32.14%)	0.000002	**0.000003**
ko04730	Long-term depression	4 (14.29%)	0.000168	**0.002455**
ko00220	Arginine biosynthesis	3 (10.71%)	0.000222	**0.002698**
ko04713	Circadian entrainment	4 (14.29%)	0.000765	**0.006978**
ko04970	Salivary secretion	4 (14.29%)	0.000765	**0.006978**
ko04371	Apelin signaling pathway	4 (14.29%)	0.000919	**0.007458**
ko04926	Relaxin signaling pathway	4 (14.29%)	0.001457	**0.010638**
ko04020	Calcium signaling pathway	4 (14.29%)	0.002850	**0.018912**
ko00330	Arginine and proline metabolism	3 (10.71%)	0.004711	**0.028656**
ko00591	Linoleic acid metabolism	2 (7.14%)	0.008181	**0.045937**
ko04726	Serotonergic synapse	3 (10.71%)	0.009297	**0.046753**
ko04750	Inflammatory mediator regulation of TRP channels	3 (10.71%)	0.009607	**0.046753**
ko04913	Ovarian steroidogenesis	2 (7.14%)	0.023420	0.106853
ko00590	Arachidonic acid metabolism	2 (7.14%)	0.035151	0.150942
ko04621	NOD-like receptor signaling pathway	2 (7.14%)	0.047399	0.186554
ko04391	Hippo signaling pathway -fly	3 (10.71%)	0.048555	0.186554

^a^ The bold values showed the 15 pathways with *Q* < 0.05 (adjusted *p*-value).

**TABLE 5:** Some of the 121 candidate genes related to fecundity.

GeneID	Symbol	Description	Pathway^a^
LVAN09598	PARD3	partitioning defective 3 homolog isoform X3 [*Cimex lectularius*]	ko04391
LVAN09729	Rere	arginine-glutamic acid dipeptide repeats protein-like isoform X1 [*Hyalella azteca*]	ko04391
LVAN11224	CYP2L1	cytochrome P450 [*Panulirus argus*]	ko00591, ko04726, ko04750, ko04913, ko00590
LVAN11225	CYP2L1	cytochrome P450 [*Panulirus argus*]	ko00591, ko04726, ko04750, ko04913, ko00590
LVAN18641	Plcb4	1-phosphatidylinositol 4,5-bisphosphate phosphodiesterase beta-2 [*Daphnia magna*]	ko04261, ko04022, ko04919, ko04730, ko04713, ko04970, ko04371, ko04926, ko04020, ko04726, ko04750, ko04621
LVAN24327	trx-2	thioredoxin, mitochondrial [*Ursus maritimus*]	ko04621

a Only the 19 significantly enriched KEGG pathways were shown.

## Discussion

Over the past 30 years, a variety of *P. vannamei* strains with specific phenotypic and genotypic characteristics have been cultivated in different artificial selection projects. Studies on Atlantic salmon have shown that even selecting the same trait may leave different marks in the genomes of different populations (Gutierrez et al., 2015a; Gutierrez et al., 2015b; López et al., 2019). The possible reason was that selection may act on different genes (Amaral et al., 2011), and similar phenotypes may form different genetic pathways among different populations (Vasemägi et al., 2012; Perrier et al., 2013; Elmer et al., 2014; Mäkinen et al., 2014; Pujolar et al., 2017). In this study, three populations were used, among which two were characterized by strong resistance and one by fast growth. To the best of our knowledge, this is the first study of genomic signatures of artificial selection in fecundity of female shrimp with pooled DNA-seq. The genomic signatures in fecundity obtained from populations with different characteristics may be less but more representative.

The combination of DNA-seq and the DNA pooling approach can associate specific regions of the genomic signatures with target traits. Using this strategy, Bertolini et al. (2016) identified genome-wide polymorphisms in two cultured European sea bass (*Dicentrarchus labrax*). Naval-Sanchez et al. (2020) analyzed the domestication of aquaculture species independently, which was the first analysis on the domestication of aquaculture species. In this study, more than 600,000 SNPs were mined for each bulk. Fst and *p* ratio were used to determine the selective sweep region. Fst was calculated using the Bayesian method, which might result in a high degree of false positives (Lotterhos and Whitlock, 2015). After the intersection of Fst and *p* ratio, only 64,046 SNPs were shared by both bulks, showing that the setting threshold was strict (*p* < 0.01). In the selective sweep regions, the shared sites exhibited a Ts/Tv ratio of 1.49 to 1.53, which is lower than another ratio reported in *P. vannamei* (2.0, Yu et al., 2014). In fish, the reported Ts/Tv ratio ranged from 0.95 to 1.49 in several *Oncorhynchus* species (Smith et al., 2005), 1.69 in *Takifugu rubripes* (Cui et al., 2014), and 1.37 in *Salmo salar* (Hayes et al., 2007). If transition and transversion randomly occurred, the Ts/Tv ratio should be 0.5, because there are two possibilities of transitions and four possibilities of transversion (Kraus et al., 2011). However, transition is usually more frequent than transversion in the genome (Rosenberg et al., 2003), which may be related to cytosine methylation (Shen et al., 1994). Different targeted regions may affect the Ts/Tv ratio, like whole-genome, exon, specific genes, which is also greatly affected by the CpG islands and GC content of the region (Dohm et al., 2008). The Ts/Tv ratio can reflect the characteristics of a targeted region of a specific genome to a certain extent.

As SNP calling was based on bioinformatics analysis of pooled DNA-seq data, it was necessary to validate the polymorphisms of SNPs at an individual level. Even though the most rigorous parameter setting was used to determine the selective sweep regions, in the subsequent SNP verification, only 6 of the 44 loci displayed significant differences in populations with much different fecundities. The SNPs had a high false-positive rate even in the offspring population, which showed genomic signatures might have strong population specificity in *P. vannamei*. If more samples were used for genotyping, the proportion would increase. The SNPs detected in this study could be used to calculate the genomic relationship matrix (GRM) and predict the genomic estimated breeding value (GEBVs) of candidate populations in the future GS programs (Luo et al., 2021), which have higher prediction accuracy than the GBLUP method.

Among the 121 identified genes in the selective sweep region, *LvPlcb4* existed in 45 of the 73 enriched KEGG pathways. It belonged to the phospholipase C (PLC) family and was a membrane-related enzyme. The bioactive substances produced by the PLC family participate in a variety of cellular processes through intercellular and extracellular signal transduction pathways (Kim et al., 2015). PLC generated the second messengers inositol 1,4,5-triphosphate (IP3) and diacylglycerol (DAG) by catalyzing the hydrolysis of PIP2 to stimulate the release of Ca^2+^ in cells. Granulosa cells indirectly regulate the development and maturation of oocytes by transmitting signals of IP3, Ca^2+^, and other second messengers to oocytes (Buccione et al., 1990; Nance, 2005). The homeostasis of Ca^2+^ is closely related to the genesis of oocytes (Zhang et al., 2010). *Lvpard3* is a homologue of partitioning defective 3 (PARD3) gene, which is a scaffold protein and plays an important role in regulating cell polarity, proliferation, migration, and tight junction (Assémat et al., 2008). Khadar found that PARD3 could promote cell proliferation and growth (Khadar and Kuo, 2018). Dong et al. observed in mice that luteinizing hormone can enhance the polarity of ovarian granulosa cells and promote cell proliferation and growth by stimulating the expression of *pard3* in ovarian granulosa cells (Dong et al., 2021). *Lvtrx2* is another important gene annotated in NOD-like receptor signaling pathway in this study. TRX2 is a member of thioredoxin (TRX) super protein family, which may be involved in the optimal growth and maturation of follicles (Damdimopoulos et al., 2002). *Lvcyp2L1* is a member of the cytochrome P450 enzyme system, is widely distributed in the endoplasmic reticulum and mitochondria in the cell membrane system, and is one of the key enzymes for steroid transformation before oocyte maturation (Boyle et al., 1998). Sakai showed that the *P450c17* gene was highly expressed in the prophase of rainbow trout ovary and oviposition (Sakai et al., 1992). In channel catfish, Kumar detected that the *P450c17* gene was at a high level in the middle stage of oocyte development (Kumar et al., 2000). It is important to note that the use of pooled samples for DNA-seq does not inherently enable the evaluation of within-group variation in expression profiles, such as inter-individual or inter-family variation. Therefore, the genes identified in this study should be considered with caution until further validation.

In conclusion, in two fecundity separated populations of *P. vannamei*, 145 selective sweep regions in the genome were identified combining two parameters, namely, Fst and *p* ratio. A total of 64,046 SNPs and 121 genes were screened from the sweep regions, which need further verification. GO annotation and KEGG enrichment analyses showed that partial genes were essential for fecundity regulation. These results would provide important clues for the differences in fecundity of female Pacific white shrimp at the genomic level.

## Data Availability

The datasets presented in this study can be found in online repositories. The names of the repository/repositories and accession number(s) can be found in the article/Supplementary Material.
